# Therapeutic tools for oral candidiasis: Current and new antifungal drugs

**DOI:** 10.4317/medoral.22978

**Published:** 2019-03

**Authors:** Guillermo Quindós, Sandra Gil-Alonso, Cristina Marcos-Arias, Elena Sevillano, Estibaliz Mateo, Nerea Jauregizar, Elena Eraso

**Affiliations:** 1 Department of Immunology, Microbiology and Parasitology, Faculty of Medicine and Dentistry, Universidad del País Vasco / Euskal Herriko Unibertsitatea (UPV/EHU), Bilbao, Spain; 2 Department of Pharmacology, Faculty of Medicine and Dentistry, Universidad del País Vasco / Euskal Herriko Unibertsitatea (UPV/EHU), Bilbao, Spain

## Abstract

**Background:**

Candidiasis is one of the most common opportunistic oral infections that presents different acute and chronic clinical presentations with diverse diagnostic and therapeutic approaches. The present study carries out a bibliographic review on the therapeutic tools available against oral candidiasis and their usefulness in each clinical situation.

**Material and Methods:**

Recent studies on treatment of oral candidiasis were retrieved from PubMed and Cochrane Library.

**Results:**

Nystatin and miconazole are the most commonly used topical antifungal drugs. Both antifungal drugs are very effective but need a long time of use to eradicate the infection. The pharmacological presentations of miconazole are more comfortable for patients but this drug may interact with other drugs and this fact should be assessed before use. Other topical alternatives for oral candidiasis, such as amphotericin B or clotrimazole, are not available in many countries. Oral fluconazole is effective in treating oral candidiasis that does not respond to topical treatment. Other systemic treatment alternatives, oral or intravenous, less used are itraconazole, voriconazole or posaconazole. Available novelties include echinocandins (anidulafungin, caspofungin) and isavuconazole. Echinocandins can only be used intravenously. Isavuconazole is available for oral and intravenous use. Other hopeful alternatives are new drugs, such as ibrexafungerp, or the use of antibodies, cytokines and antimicrobial peptides.

**Conclusions:**

Nystatin, miconazole, and fluconazole are very effective for treating oral candidiasis. There are systemic alternatives for treating recalcitrant infections, such as the new triazoles, echinocandins, or lipidic presentations of amphotericin B.

** Key words:**Oral candidiasis, antifungal treatment, azoles, echinocandins, fluconazole, miconazole, nystatin.

## Introduction

Oral candidiasis (candidosis) is one of the most common opportunistic buccal infection that is caused by *Candida albicans* and other species included in the genus *Candida*. Candidiasis commonly presents as a mild disease of the oral mucous membranes, but sometimes can be recalcitrant to treatment or become relapsing or recurrent. This oral infection is more frequent in people with extreme ages, or suffering from very diverse underlying diseases and, above all, in patients with immunodeficiency. Although more than 150 species of Candida have been described, 95% of oral candidiasis are caused by C. albicans. Other species, such as* Candida glabrata, Candida tropicalis, Candida parapsilosis, Candida krusei, Candida dubliniensis* or *Candida guilliermondii* can cause infections sporadically often complicating the management of these candidiasis (1-5).

**Figure 1:**
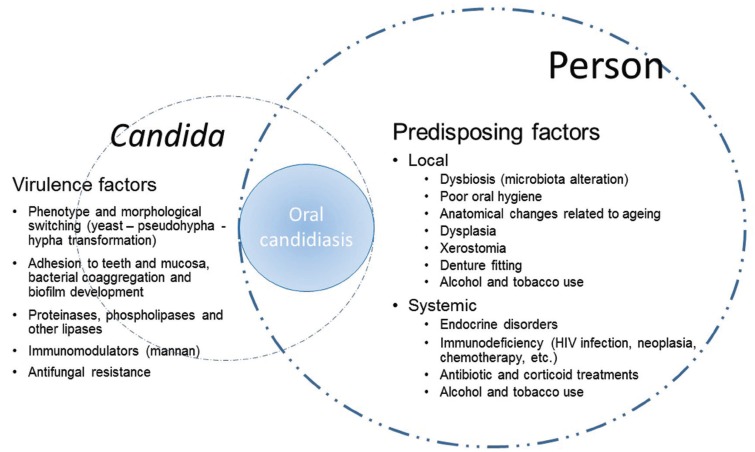
Host predisposing factors and virulence factors of *Candida* involved in the pathogenesis of oral candidiasis.

*Candida* can be part of the human oral microbiota of up to 75% of persons without known underlying diseases. This colonization occurs from birth and is greatest in the extreme ages of life (infants, children and the elderly). In adults, colonization is favoured by the use of removable dentures, in which biofilms of difficult eradication are formed, or by the presence of oral alterations, such as xerostomia, leucoplakia, lichen, etc. A greater colonization can be observed in patients who have received antibiotics, corticoids or chemotherapy, or in patients suffering from diabetes, hospitalized patients and people infected by the human immunodeficiency virus (HIV). The alteration of the balance between *Candida* and the host due to undesired changes in oral microbiota (dysbiosis) or to the damage of anatomical and physicochemical barriers facilitates candidiasis. The development of candidiasis will depend on both the virulence factors of *Candida* and the clinical conditions of the patient (Fig. 1) (1,6-8). Oral candidiasis can be classified into acute, chronic and *Candida*-associated lesions, such as angular cheilitis, denture stomatitis and median rhomboid glossitis. Acute candidiasis is dominated by pseudomembranous and erythematous candidiasis, which may become chronic. Another chronic presentation is hyperplastic candidiasis (1,4,9). Prevalence and incidence of all forms of oral candidiasis have increased in recent decades. Candidiasis are observed more frequently in patients of extreme ages, with alterations of the skin-mucous barriers by surgical interventions, receiving parenteral nutrition, treated with broad-spectrum antibiotics or/and corticoids, and presence of neutropenia or immunodeficiency. More than half of people who wear removable dentures suffer from oral candidiasis. Pseudomembranous oral candidiasis occurs between 1% and 30% of infant and children and even its prevalence is higher in patients with cancer (7-60%) or suffering from AIDS (more than 90%) (1,2,6-8,10-12).

Clinical recognition of the oral lesions by the professional is the essential foundation for diagnosis of oral candidiasis. This clinical diagnosis of oral candidiasis should be confirmed by microscopic observation of *Candida* in the appropriate clinical specimens. Moreover, *Candida* isolation and quantification in pure culture will allow a definitive identification. *In vitro* antifungal susceptibility testing is an important tool for assessing the best management of patients who have received previous antifungal treatments, who suffer relapsing infections and when candidiasis are caused by species different to *C. albicans*. However, there are still many controversial issues in the microbiological diagnosis particularly in denture stomatitis and other *Candida*-associated lesions that need to be solved (9,11,13).

## Material and Methods

We searched PubMed limiting the studies in human beings published in English and Spanish from 2010 to the date of submission, 2019. The following key words were used: “Oral candidiasis” or “oral candidosis” and “antifungal drugs”, “oral candidiasis” or “oral candidosis” and “antifungal treatment”, “oral candidiasis” or “oral candidosis” and “antifungal therapy”, “oral candidiasis” or “oral candidosis” and “polyene”, “oral candidiasis” or “oral candidosis” and “nystatin”, “oral candidiasis” or “oral candidosis” and “amphotericin B”, “oral candidiasis” or “oral candidosis” and “azole”, “oral candidiasis” or “oral candidosis” and “miconazole”, “oral candidiasis” or “oral candidosis” and “fluconazole”, “oral candidiasis” or “oral candidosis” and “clotrimazole”, “oral candidiasis” or “oral candidosis” and “isavuconazole”, “oral candidiasis” or “oral candidosis” and “itraconazole”, “oral candidiasis” or “oral candidosis” and “posaconazole”, “oral candidiasis” or “oral candidosis” and “echinocandins”, “oral candidiasis” or “oral candidosis” and “anidulafungin”, “oral candidiasis” or “oral candidosis” and “caspofungin”, and “oral candidiasis” or “oral candidosis” and “micafungin”. We also searched the Cochrane Library (https://www.cochranelibrary.com). We excluded letters and abstracts of meetings.

## Results

**Figure 2:**
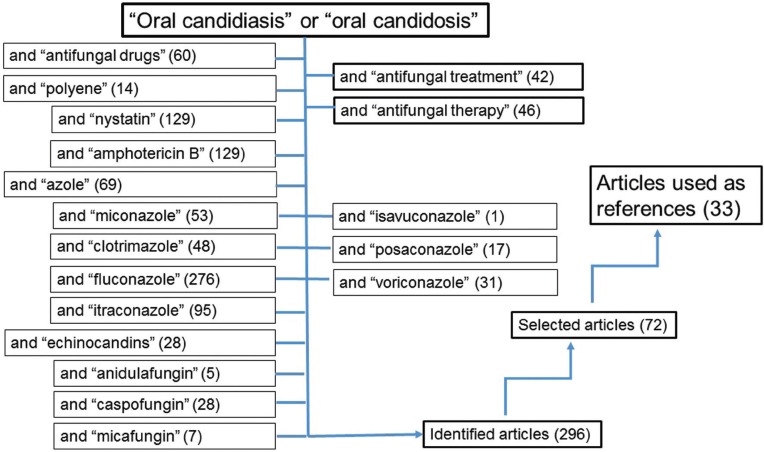
Flow diagram showing the key words used in the bibliographic search. Number of studies are indicated in parenthesis.

The bibliographic search identified 296 articles, of which 72 were selected after reading the manuscripts summaries (Fig. 2). Following the analysis of these articles, 33 manuscripts were finally included in the references, after excluding those publications that did not fit the aims of the present review.

## Discussion

Treatment of oral candidiasis is based on three foundations: Early and accurate diagnosis of the type of oral candidiasis, correction of the predisposing factors or underlying diseases, and the use of the most appropriate antifungal drugs. Promotion of good oral hygiene and periodic oral examination, controlling predisposing or facilitating factors, are fundamental to prevent infection facilitate treatment if they occur. The choice of antifungal drug should take into account the patient immune status, the specific characteristics of oral candidiasis (clinical presentation, aetiology, susceptibility to antifungal drugs, organic location, dissemination) and the pharmacological characteristics of the available antifungal drugs (administration, metabolism, elimination, interactions with other drugs and toxicity). Three large families group the most commonly used antifungal drugs: polyenes (amphotericin B and nystatin), echinocandins (anidulafungin, caspofungin and micafungin) and azoles. Azoles constitute the most extensive group being divided into imidazoles (clotrimazole, miconazole, ketoconazole, etc.) and triazoles (fluconazole, isavuconazole, itraconazole, posaconazole and voriconazole) ( Table 1, Table 2). Other drugs with different antifungal actions and possible systemic use against superficial mycoses, such as flucytosine, griseofulvin and terbinafine, are not used commonly in oral candidiasis (7,8,11,14-17). Finally, other therapeutic alternatives under development involve the use of new antifungal drugs, terpens, probiotics, peptides with antifungal activity, sera with polyclonal or monoclonal antibodies or cocktails of cytokines (18-25).

**Table 1:**
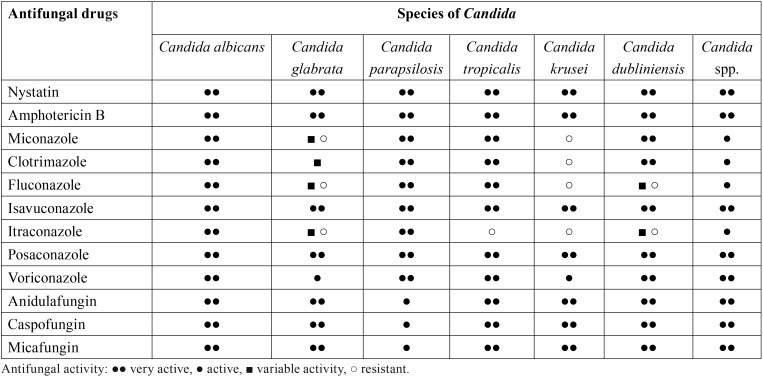
*In vitro* activity of the main antifungal drugs against main *Candida* species causing oral infection.

**Table 2:**
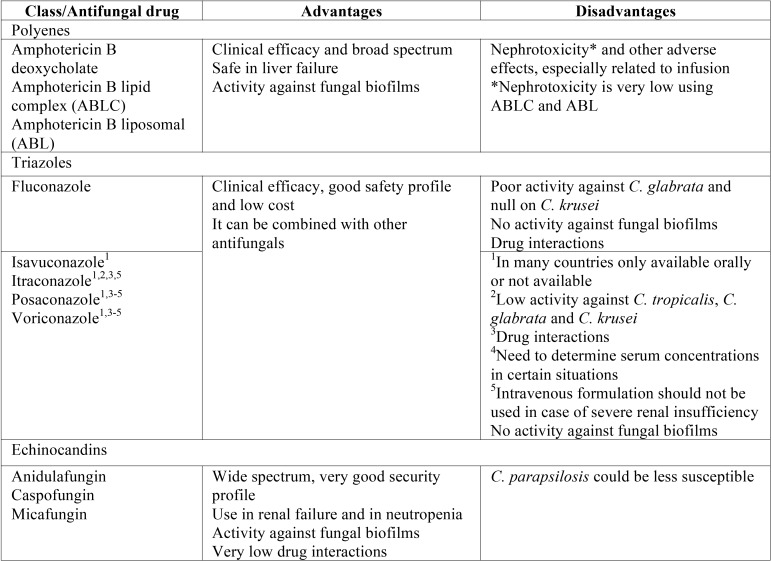
Antifungal drugs available for systemic use in the treatment of oral candidiasis.

**Figure 3:**
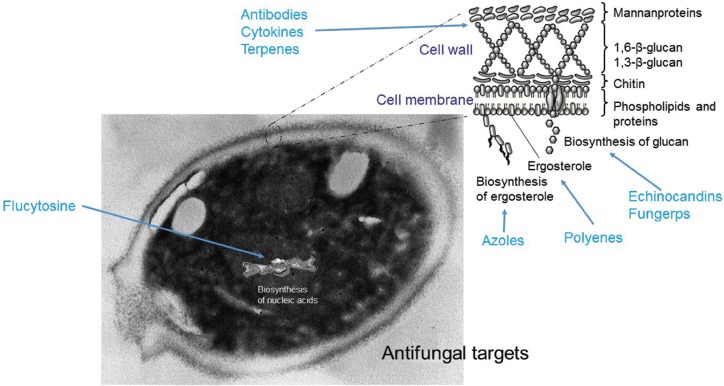
Fungal targets of current and new antifungal drugs.

The main mechanisms of antifungal action consist in the alteration of the membrane or the fungal cell wall by inhibition of molecules essential for these, such as ergosterol (azoles) or 1,3-ß-D-glucan (echinocandins), or by binding to ergosterol (polyenes), causing the formation of pores and altering the integrity and permeability of the cell membrane (Fig. 3). The actions of polyenes and echinocandins are usually fungicidal. Conversely, azoles are fungistatic for *Candida* at therapeutic doses (7,8,26-28).

Antifungal treatment of oral candidiasis can be carried out topically or systemically, usually with oral formulations. Topical drugs are applied to the affected area and treat limited infections. Systemic drugs are prescribed when the infection is more widespread and has not been enough with the topical therapy. Topical antifungals have few and mild adverse effects because their absorption is very limited, and do not interact with other drugs the patient may be receiving. The efficacy of topical agents in the treatment of oral mycoses depends on the type and size of the lesion, the mechanism of action of the drug and the characteristics of the formulation, such as viscosity, hydrophobicity and acidity. Antifungal formulations are marketed as oral suspensions, tablets, pastilles, gels, mucoadhesive tablets, toothpastes, etc. for facilitating their therapeutic action that are very effective in curing most oral candidiasis in a few weeks (15,17,29).

Although systemic azoles and echinocandins, with better tastes and less gastrointestinal adverse reactions, have provided new clinical options, topical therapy using nystatin (polyene) and miconazole (azole) are still the main recommended treatments for oral candidiasis due to its high efficacy, low cost, and less side effects, especially in low-income countries. The accumulated experience is important and their usefulness is clearly defined (7,8,14-17).

In 2016, the Infectious Diseases Society of America (IDSA) updated its clinical practice guidelines for the management of candidiasis, including oral candidiasis (11). These recommendations include the treatment of mild disease with miconazole (muco-adhesive buccal 50-mg tablet once daily for 7-14 days). Alternatives for mild disease include nystatin suspension (100,000 U/mL, 4-6 ml, four times daily) or pastilles (1-2 pastilles, 200,000 U each, four times daily for 7-14 days). Moreover, the World Health Organization recommended that topical therapy with nystatin suspension or pastilles can be an alternative to oral fluconazole for treating oropharyngeal candidiasis in HIV-positive children and adults (8).

Nystatin, obtained from *Streptomyces noursei*, binds to the ergosterol of the fungal plasma membrane and forms pores that make it more permeable, causing a loss of intracellular potassium with a fungicidal effect. Moreover, nystatin also causes secondary cell damage by autooxidation. The anti-*Candida* spectrum of nystatin is quite broad. The antifungal cut-off point for polyenes in the in vitro susceptibility tests is a minimum inhibitory concentration (MIC) of 1 μg/ml. Some clinical isolates resistant or less susceptible have been described in *Candida lusitaniae, Candida rugosa, Candida haemulonii, Candida lipolytica, C. guilliermondii, C. krusei* and *C. glabrata*. Nystatin is not absorbed orally. Its parenteral use is toxic, but clinical trials have been conducted with a liposomal formulation that allows its intravenous administration. Its undesirable effects include local irritation, and when administered orally, nausea, vomiting and diarrhoea. Nystatin is probably safe during pregnancy and breastfeeding. Treatment is effective only if nystatin is administered over a sufficient period. However, the unpleasant taste and this prolonged treatment pattern compromise compliance by the patient (11,14).

The meta-analysis of Lyu X *et al.* (8) showed that nystatin suspension and pastilles in combination for two weeks achieved a higher clinical and mycological cure rates, and using the nystatin pastilles alone might have a higher mycological cure rate, when compared with using nystatin suspensions alone. Nystatin pastilles at a dose of 400,000 IU resulted in a significantly higher mycological cure rate than that administrated at a dose of 200,000 IU. Furthermore, treatment with nystatin pastilles for four weeks seemed to have better clinical efficacy than treatment for 2 weeks. Nystatin suspension was not a good choice for infants, children, and HIV/AIDS patients with oral candidiasis, probably because of its short-term action on the oral mucosa. Moreover, exposure to nystatin at a concentration 0.25 to 1 times the MIC value for 30 minutes resulted in a beneficial post-antifungal effect, the delay in fungal regrowth that persists after a brief exposure to an antifungal agent. (8). Encapsulation of nystatin in nanoparticles or the inclusion in toothpastes or tissue conditioners exhibit properties that enable its *in vitro* functionality and may provide the basis for new successful approaches for the treatment of oral candidiasis (20,30,31).

Amphotericin B is other polyene that has been used for many years in the treatment of oral candidiasis, but at present, it is practically impossible to find topical preparations of this antifungal drug in many countries, including Spain. IDSA recommends amphotericin B deoxycholate oral suspension as alternative for fluconazole-refractory oral candidiasis (11). In these severe situations, amphotericin B deoxycholate, the conventional intravenous formulation, and the less nephrotoxic formulations of amphotericin B liposomal and amphotericin B lipid complex can be used (26).

Miconazole and the rest of azoles (imidazoles or triazoles) block the fungal cytochrome P450-dependent lanosterol 14-α-demethylase (Erg11p). This action is fungistatic, causing an important alteration of the cellular permeability and the inhibition of the growth of the fungal cell. Azoles develop their effect more slowly than polyenes, but have less toxicity because their action against fungal membranes is more selective than that of polyenes. Miconazole has a good *in vitro* antifungal activity against *Candida* but this activity is lower against some isolates of *C. glabrata* and *C. krusei*. Miconazole can be administered topically, orally or intravenously, but these latter two ways are very infrequent. It is administered as miconazole buccal tablets, miconazole chewing gum, miconazole oral gel, and miconazole lacquer. In some countries, there is an alternative presentation of miconazole as one-daily 50-mg mucoadhesive buccal tablet (32). Once daily application is an advantage over applying nystatin four or five times a day to maintain patient compliance. Miconazole mucoadhesive tablets exhibited higher salivary concentrations and better tolerance for the patient. The recent meta-analysis of Zhang LW *et al.* (15) showed that miconazole was more effective than nystatin for pseudomembranous oral candidiasis. Single daily dose regimens of miconazole buccal tablets are useful for poorly compliant patients. However, in HIV-infected patients, there was no significant difference in the efficacy between miconazole and other antifungal drugs. There was no significant difference between miconazole and other antifungals in terms of the relapse rate. Despite its good properties, topical use of miconazole can cause itching or burning sensation due to local irritation (less than 5% of patients). In addition, miconazole has the drawback of possible interaction with other drugs, such as warfarin because inhibits the enzyme cytochrome P450. Hellfritzsch M *et al.* (33) found evidence supporting a clinically relevant drug-drug interaction between warfarin and miconazole oral gel. In contrast, these authors did not find any indication of an interaction between warfarin and nystatin oral solution.

Other imidazoles for topical use are bifonazole, clotrimazole, eberconazole, fenticonazole, flutrimazole, oxiconazole, sertaconazole, sulconazole, terconazole, and thioconazole, with a broad-spectrum including *Staphylococcus epidermidis* and other Gram-positive bacteria. However, most are not marketed for oral use in many countries, including Spain.

Triazoles, such as fluconazole, isavuconazole, itraconazole, voriconazole and posaconazole, have a broader spectrum and they are used for the systemic treatment of many severe mycoses. There are currently oral and intravenous formulations but some of them are not available in many countries. One of the main disadvantages is that inhibit various isoforms of cytochrome P450, which causes an inhibition of the metabolism of other drugs metabolized by this route, thus increasing their plasma concentrations. This occurs with immunosuppressant drugs (cyclosporine, tacrolimus, sirolimus, etc.), oral anticoagulants (warfarin), some statins, H1 antihistamines, benzodiazepines, HIV infection, protease inhibitors, and calcium channel blockers. In addition, other drugs, such as rifampicin, phenytoin, carbamazepine, H2 antihistamines, proton pump inhibitors and some antacids, when administered together with azoles, can reduce plasma concentrations of these. They are category C drugs and you have to avoid them in pregnancy if there are other alternatives.

Oral fluconazole (100-200 mg daily for 7-14 days) is recommended for treating moderate to severe oral candidiasis (11). Chronic suppressive therapy is usually unnecessary for oral candidiasis. If required for patients who have recurrent infection, fluconazole, 100 mg 3 times weekly, is recommended. Fluconazole has a good antifungal activity against most of the species of *Candida*. For the clinical isolates of *albicans, parapsilosis* and *tropicalis*, the *in vitro* susceptibility cut-off point is 2 μg/ml. In contrast, for isolates of *glabrata*, an MIC of fluconazole less than 32 μg/ml indicates a dose-dependent susceptibility, whereas all isolates of *C. krusei* are considered intrinsically resistant to fluconazole, independently of the MIC. Infections caused by isolates of *Candida glabrata* with dose-dependent susceptibility to fluconazole can often be treated satisfactorily using doses of 800 mg/day or more (11). Fluconazole resistances have been described in some isolates of *C. albicans* and *C. dubliniensis* from HIV-infected patients with repeated episodes of oropharyngeal candidiasis treated with fluconazole. The maximum activity of fluconazole against *Candida* is reached from a value of AUC24h/MIC of 25-100. Fluconazole is characterized by its excellent bioavailability and low toxicity. The incidence of adverse effects with fluconazole is low, among which the most frequent are nausea, vomiting, headaches, rash, abdominal pain and diarrhoea. Serious side effects are very rare.

In fluconazole-refractory disease, itraconazole oral solution (200 mg once daily for up to four weeks) is recommended. Itraconazole is a first generation lipophilic triazole of limited used because of its irregular oral absorption and its pharmacological interactions. It is active against many clinical isolates of *C. glabrata* and *C. krusei* resistant to fluconazole. In the case of clinical isolates of *Candida*, the *in vitro* susceptibility cut-off point is 0.125 μg/ml. The maximum activity against *Candida* is reached from an AUC24h/MIC index of 25-100. The oral formulation can cause gastric discomfort. There is a transient elevation of transaminases in about 2% of patients. Neuropathy, hallucinations, cerebellar alterations and hypertriglyceridemia have also been observed. It is contraindicated in patients with heart failure because of its negative inotropic effect. Oude Lashof AM *et al.* (34) compared in one randomized study, the efficacy of fluconazole (100 mg/day for 10 days) and itraconazole (200 mg/day for 15 days) in cancer patients with oropharyngeal candidiasis. The fluconazole group got a clinical and mycological improvement of 66% compared to 54% for the group treated with itraconazole. Fluconazole had a significantly better clinical and mycological cure rate compared with itraconazole.

Posaconazole suspension (400mg twice daily for three days followed by 400mg daily for up to four weeks) is an alternative for treating fluconazole-refractory oral candidiasis. Posaconazole has a structure similar to that of itraconazole and shows one of the widest antifungal spectrum of all triazoles. The *in vitro* susceptibility cut-off point is 0.06 μg/ml and the maximum activity of posaconazole against *Candida* is reached from an AUC24h/MIC index of 25-100 of posaconazole. Its binding to plasma proteins is very high (98-99%). The most frequent adverse effects are gastrointestinal discomfort, rash, headache and alterations in the electrocardiogram (prolongation of the QT segment) (5,11,25).

Voriconazole is a second-generation triazole developed from fluconazole with also a broad antifungal spectrum that can be used as alternative for the treatment of fluconazole-refractory oral candidiasis. The *in vitro* susceptibility cut-off point is 0.125 μg/ml for the clinical isolates of *C. albicans, C. parapsilosis* and *C. tropicalis*; however, it has been established at 0.5 μg/ml for *C. krusei* (35). The maximum activity against *Candida* is reached from an AUC24h/MIC index of 25-100. It is widely distributed throughout the tissues and organs. Its fixation to plasma proteins is 60%. The oral formulation can cause gastric discomfort. Transient visual disturbances have also been described in one third of patients, abnormalities in liver enzymes that sometimes lead to suspension of treatment, rash, hallucinations, headache, confusion, hypotension and haematological alterations. Exceptional cases of clinical hepatitis, cholestasis and fulminant hepatic failure have been reported (11,25,36).

Finally, isavuconazole is a new azole, structurally related to fluconazole and voriconazole, with one of the widest antifungal spectrum of all triazoles that presents a very high oral absorption not interfered by the presence of food, gastric pH modifications, or mucositis. Its distribution volume is very high, in spite of it is highly bound to plasma proteins. The pharmacological interaction with other drugs seems to be lower compared to other azoles, facilitating the management of these interaction, which is probably the most important advantage of this antifungal drug (37).

Echinocandins are a family of semisynthetic lipopeptides with a highly selective target, the biosynthesis of 1,3-ß-D-glucan of the fungal wall, by blocking the activity of the enzyme beta-glucan synthetase, with fungicidal effect against *Candida* and few toxic effects for human eukaryotic cells. Its use is exclusively intravenous. The cut-off points of echinocandins are set at a MIC of 0.125 μg/ml for *C. glabrata* (except for micafungin, for which it is 0.06 μg/ml), of 0.25 μg/ml for *C. albicans, C. krusei* and *C. tropicalis*, and 2 μg/ml for *C. guilliermondii* and *C. parapsilosis*. The pharmacodynamics indicator that is related to therapeutic success in the treatment of candidiasis is Cmax or AUC24h over MIC. The maximum activity against *Candida* would be achieved with serum concentrations of the free drug four times higher than the MIC (Cmax/MIC ≥ 4) or with an AUC24h/MIC value ≥ 20. Among the advantages of echinocandins for the treatment of severe and recalcitrant oral candidiasis are their anti-biofilm activities and their prolonged post-antifungal effect. They can be first choice drugs for the treatment of severe candidiasis in patients with immunodeficiency, the seriously ill and those with a high probability of drug interactions. They are category C drugs in pregnancy and should be avoided if there is another therapeutic alternative, as during breastfeeding (38,39,40).

There are other pharmacological preparations that have antifungal activity and can be used in combination with antifungal drugs. These formulations include chlorhexidine, povidone iodine, solutions of gentian violet, potassium permanganate, methylene blue or sodium hyposulfite, propylene glycol, selenium sulphide, boric acid, and caffeic acid derivatives (17,21). Several investigational agents are currently under development against old targets, such as ergosterol (tetrazoles VT-1129, VT-1161, and VT-1598) or 1,3-β-D-glucan (extended half-life echinocandin CD101 or ibrexafungerp -SCY-078-) that may offer advantages over currently available drugs. Ibrexafungerp has the advantages of its oral and intravenous administration and of being active against fluconazole- and echinocandin-resistant oral isolates of *Candida*. Several agents with novel mechanisms of action are also under development such as inositol acyltransferase AX001, the dihydroorotate dehydrogenase inhibitor F901318, and VL-2397. These newer antifungal drugs may be less susceptible to mechanisms of antifungal resistance. Each of these investigational agents has the potential to improve patient outcomes in the treatment of oral candidiasis. (25).

Treatment of oral candidiasis has several tools available to be successful. Topical treatments with nystatin or miconazole are effective in most infections and systemic treatment with oral fluconazole shows a similar efficacy. The treatment must be personalized considering the different clinical characteristics of the patient to avoid physiological interactions (pregnancy, lactation, etc.) or pharmacological (elderly with multiple treatments, critical patients, patients with neoplastic pathologies or immunodeficiency, etc.). In the face of treatment failures, recurrent or relapsing infections, we can count on new antifungal agents with old and new mechanisms of action. Among these drugs, the new triazoles, and echinocandins offer promising alternatives for treatment.
